# X-ray Fluorescence Analysis of Pigments in Gothic Frescoes, Coats of Arms, and Polychromy on Sculptures on the Triforium in St. Vitus Cathedral on the Territory of the Czech Kingdom in the 14th Century

**DOI:** 10.3390/ma15145033

**Published:** 2022-07-20

**Authors:** Tomáš Čechák, Tomáš Trojek, Vladimír Růžek, Radka Šefců, Hana Průšová

**Affiliations:** 1 Department of Dosimetry and Application of Ionizing Radiation, Faculty of Nuclear Science and Physical Engineering, Czech Technical University in Prague, Břehová 7, 11519 Prague, Czech Republic; tomas.trojek@fjfi.cvut.cz (T.T.); v.ruzek1918@gmail.com (V.R.); hana.prusova@fjfi.cvut.cz (H.P.); 2 National Gallery in Prague, Staroměstské Nám. 12, 11015 Prague, Czech Republic; radka.sefcu@ngprague.cz

**Keywords:** X-ray fluorescence analysis, coats of arms, pigments, characteristic radiation, St. Vitus Cathedral

## Abstract

Pigments in the paints used for the coats of arms, polychromy on sculptures, and pigments in frescoes characterize not only the epoch but also individual creators or groups of artists involved in their creation. X-ray fluorescence analysis is a non-destructive method suitable for determining the chemical composition of these artifacts. This article covers the results of measurements of selected objects, and compares them with similar objects from the territory of the Czech Kingdom in the 14th century.

## 1. Introduction

**Figure 1:**
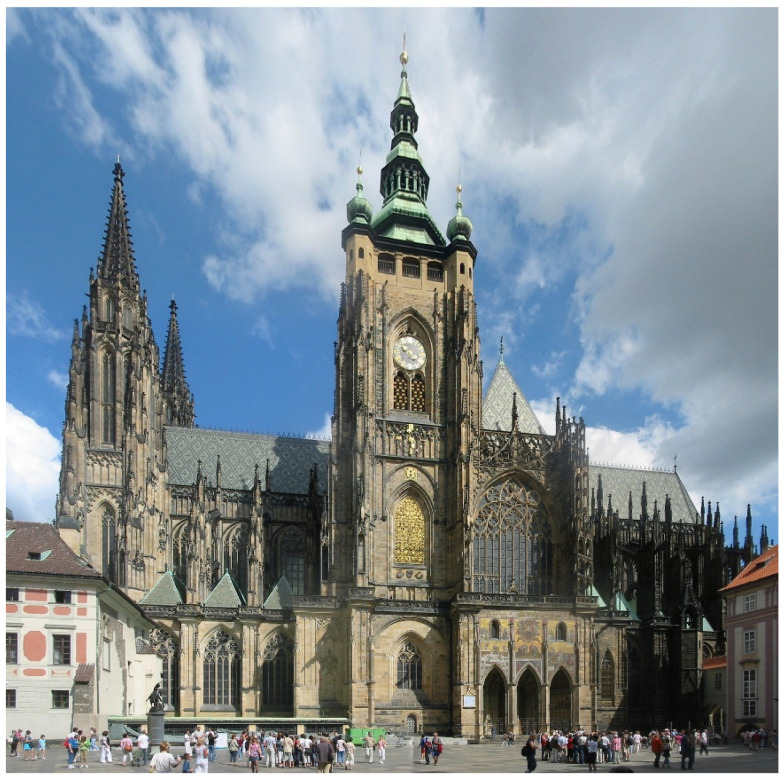
St. Vitus Cathedral [4].

The gallery with portraits of the Luxembourg family, which ruled the Czech Kingdom from 1310, the clergy of the Prague archbishopric, and the directors and architects of St. Vitus Cathedral from the late 1470s, is a unique work of art on a European scale. The bust of the builder, Petr Parléř, is the first author’s self-portrait in European sculpture. The entire gallery in the lower inner gallery of the peristyle, in the so-called triforium of St. Vitus Cathedral, is the work of the Parléř construction plant. Most of the busts are accompanied on the left and right sides by appropriate plastic coats of arms, which are painted in color. The individual busts are also painted in color. St. Vitus Cathedral is the most important Czech Gothic cathedral and the dominant feature of Prague Castle, see Figure 1. Construction began at Prague Castle in 1344, and on the site of the Prague Bishop’s residence in the 10th century. In 1375, the choir of the cathedral of St. Vitus planted triforium stones. Busts in the choir polygon, dedicated to Charles IV and his family, were made out and their place was determined before the death of Charles IV on 29 November 1378. The bust of Václav of Luxembourg and Brabant (* 1337-† 1383) was the first to be placed on the longitudinal side of the triforium. Other portrait busts on the longitudinal sides of the triforium were installed in the years 1379–1380. This time frame for the creation of the busts also determines the time when the coats of arms and busts were painted, up to the years 1379–1380 [1]. The construction of the cathedral was interrupted at the beginning of the 15th century and was not definitively completed until the 20th century. The busts were damaged in 1620 during the Thirty Years’ War. The measurements that we made aimed to determine the composition of the pigments that were used, to distinguish medieval pigments from repainting and retouching, and to compare the pigments used with the pigments used on frescoes and coats of arms in the 14th and 15th centuries. In the St. Vitus Cathedral, in the Vlašim Chapel, there are three coats of arms on the wall behind the tomb of Archbishop Jan Očko (+1380). The composition of the pigments was also measured on these coats of arms. Individual statues and other decorations of St. Vitus Cathedral have been examined in detail in the past, such as the statue of St. Wenceslas in the Wenceslas Chapel [2]. Part of the decoration has already been restored; for example, murals in the St. Wenceslas Chapel [3]. Statues and coats of arms on the triforium have not yet been studied, despite their undeniable artistic significance. The coats of arms and murals in the Vlašim Chapel in St. Vitus Cathedral have also not been examined. The sculptural work of the second builder of the cathedral, Petr Parléř (1356–1399), was the source of inspiration for the so-called “Beautiful Style” (Weicher Stil), which influenced the whole of central Europe, including Prussia, Silesia, and Austria. Thanks to its beautiful style, Prague remained until the end of the 14th century at the forefront of European art.

## 2. Materials and Methods

Pigments on busts and coats of arms were analyzed non-destructively, by X-ray fluorescence analysis. This is an instrumental analytical method, which uses characteristic radiation to determine the presence and the amount of each element in the examined sample. When an electron is ejected from the inner shell by the absorption of a photon (interaction photoeffect), the atom becomes ionized and the ions are left in a high-energy state. If this electron vacancy is on the K shell and is filled by an electron coming from L level, the transition is accompanied by the emission of an X-ray line known as the Kα line. The K shell vacancy may be filled by an electron coming from M level that is accompanied by the emission of the Kβ lines. If the electron vacancy is on the L shell and is filled by an electron coming from higher levels, the transition is accompanied by the emission of lines from series of L-lines. During the measurement, a collimated beam of X-ray radiation hits the examined area and interacts with the surface of the sample. Characteristic radiation is excited in the sample, the energy of which is given by atomic numbers of the elements present and which determines the binding energy of electrons on particular shells. By analyzing the measured characteristic radiation spectrum, we obtain information about the chemical composition of the sample. X-ray fluorescence analysis, like other spectrometric methods, is a relative method, and quantitative analysis requires exact calibration. A disadvantage of the method is that the characteristic radiation of low-Z elements has relatively low energy (less than 2 keV) [5]. Detection of such low-energy radiation requires a super-thin window and a vacuum, or at least a special atmosphere between the surface of the sample under investigation and the detector window. For the measurements, we used a handheld Niton XL3t GOLDD Plus analyzer, which does not allow for measuring the characteristic radiation of elements with Z < 12–14. This means that another method would have to be used to determine the composition of pigments of organic origin.

The analysis was performed non-invasively using the “Main” mode with a spot diameter of 8 mm, an Al/Fe filter, 50 kV X-ray tube voltage, and current of 40 μA. The measurement time was 30 s with a sample device distance of 5 mm. The measurement was performed contactless, and there was no possibility of damaging the object. The non-painted material was measured prior to the analysis to recognize background elements present in the analyzer (shielding, Ag anode, filter) and in the non-painted material. Device calibration was performed automatically.

The authors have a device for confocal micro-beam X-ray fluorescence analysis (confocal micro-XRF). This is a non-destructive analytical tool for investigating the composition of the sample that enables three-dimensionally resolved information to be acquired and the measured depth profiles to be evaluated [6]. In the sample that was analyzed, the pigments were in one layer only, so the use of the Niton analyzer was fully justified.

## 3. Results and Discussion

The basic heraldic colors include red, blue, black, and green, as well as white and yellow, which represent metals—white silver, and yellow gold. As a rule, the colors symbolizing the metals were not laid on top of each other, but on a colored base, and the metals and the other colors were combined. In exceptional cases, purple, skin tone, orange, and purple were still used in a later period [7]. On the coats of arms and busts on the triforium, we find only red, blue, black, white (silver), and yellow (gold), and on the coats of arms above the tomb of Jan Očko in the Vlašim Chapel only red, black, and yellow (gold).

Apart from the peaks indicated in the spectra, the effect of the Al/Fe filter and the detection of backscattered radiation could be observed as a signal decrease between 5 keV and 16 keV. In addition, peaks that originated in the device, such as the Ag characteristic and Compton-scattered radiation around 20–25 keV, are not indicated in the spectra. These peaks and effects can be observed in all presented spectra.

**Figure 2:**
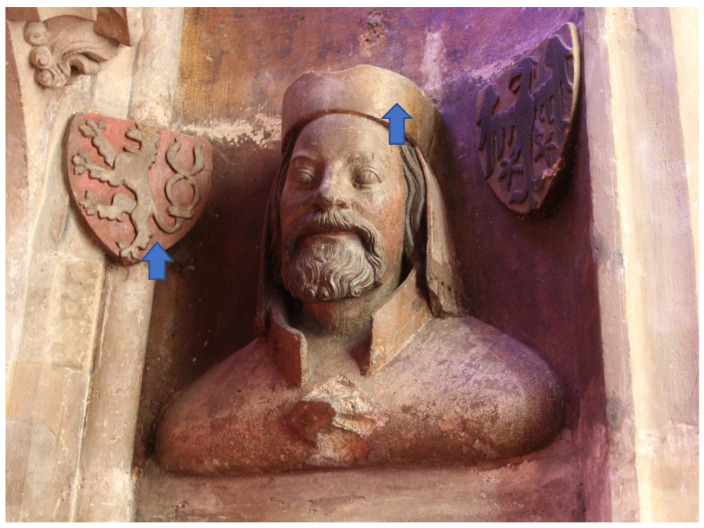
Bust—Charles IV, arrows indicate measured positions.

**Figure 3:**
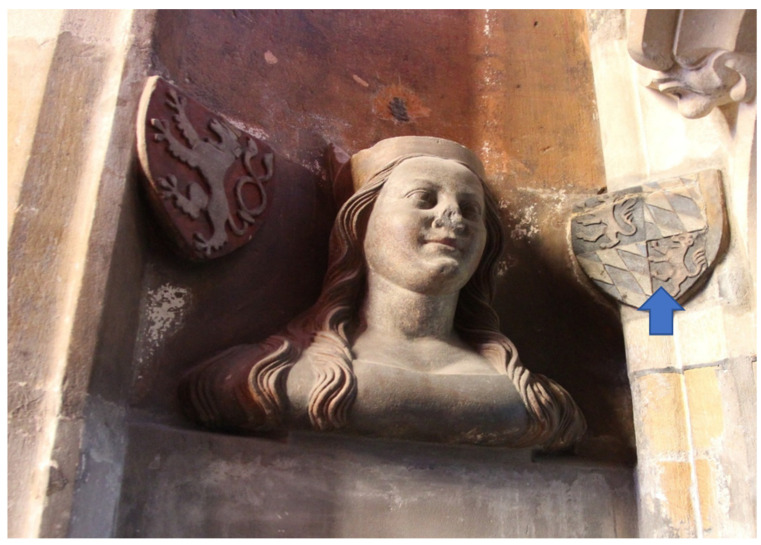
Bust—Anna Palatinate (Anna Pfalz), arrow indicates measured position.

Individual busts on the triforium are always accompanied by two coats of arms, see Figure 2 and Figure 3 The preserved pigments on the busts and on the coats of arms were both measured. In total, pigments were measured at 66 locations on the 21 busts.

**Figure 4:**
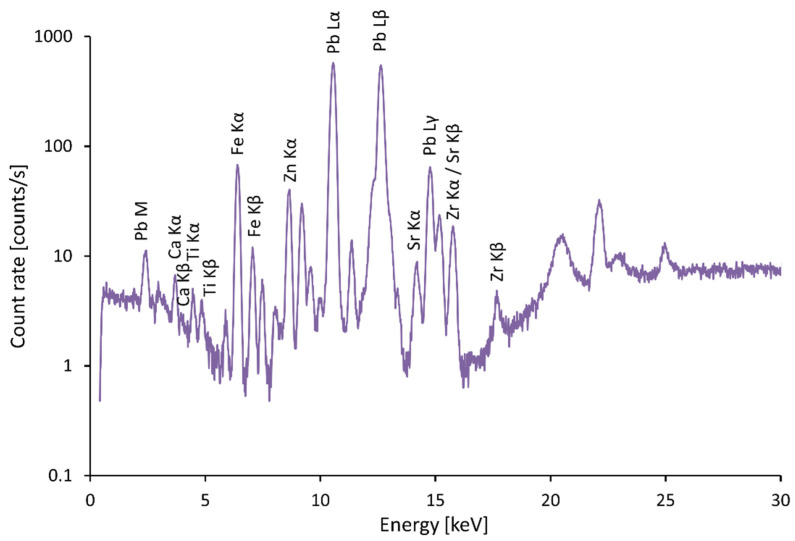
Spectrum of red lead.

**Figure 5:**
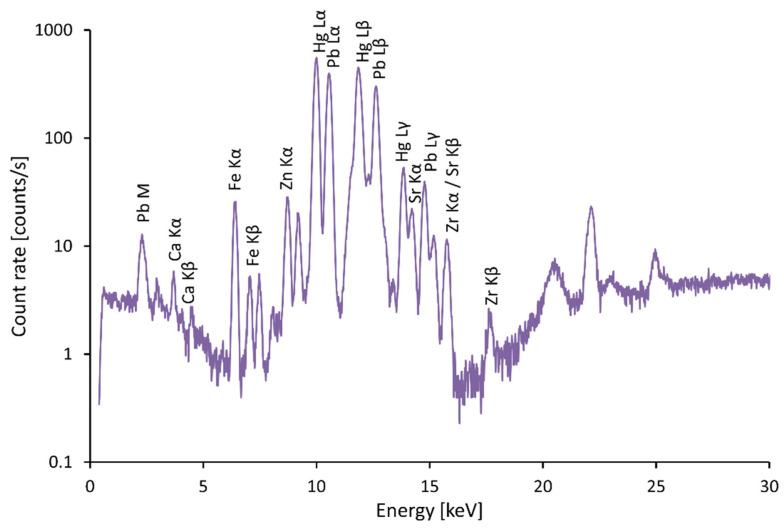
Spectrum of cinnabar, Coat of arms in right bust of Charles IV.

In most cases, red color appears on coats of arms. The most commonly used powder in the Middle Ages was red lead Pb_3_O_4_ [8]. Figure 4 is a typical spectrum of red lead. In many cases, cinnabar (HgS), also known as vermilion, was blended into the slag. In some cases, cinnabar was the main component of the pigment that was used. A combination of these two pigments affects the color and the resulting tonality of the surface and allows the desired shade to be obtained [9]. Accordingly, peaks of Hg can be observed in the spectrum, see Figure 2 and Figure 5. Vermilion was an expensive pigment, which in other frescoes was reserved only for the most important figures in the central part of the frescoes. In the frescoes at the chateau in Žirovnice, which belonged to a rich tenant of silver mines in Kutná Hora, vermilion was therefore used only in one case on the central fresco created by Italian masters at the end of the 15th century, on the robe of Christ the Judge. Red clays of domestic origin were used on other figures [10]. On such an important building as St. Vitus Cathedral, the use of cinnabar is common.

**Figure 6:**
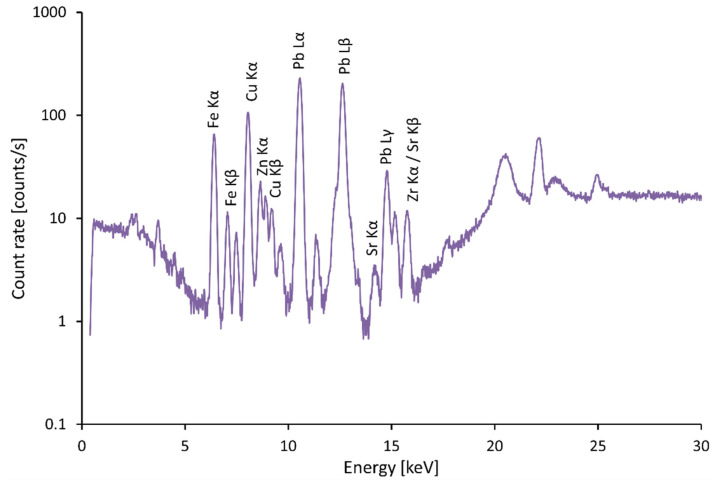
Spectrum of azurite, Coat of arms in left bust of Anna Palatinate (Anna Pfalz).

**Figure 7:**
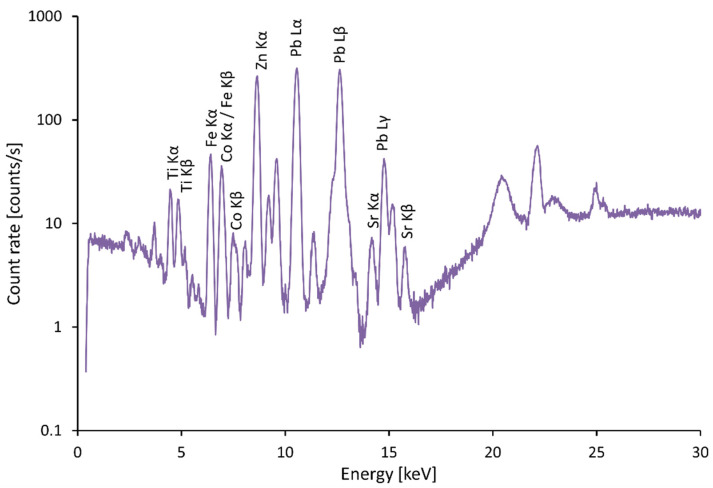
Presence of cobalt blue in blue color, retouched place.

Azurite was used as a blue pigment in the Middle Ages. Cu(OH)_2_·2CuCO_3_ is present, e.g., on the coat of arms of the Rhine palatinate on the left side of the bust of Anna Palatinate (Anna Pfalz), see Figure 5 and Figure 6. The presence of Co in the blue pigment indicates the presence of cobalt blue CoO·Al_2_O_3_, which began to be used only in the 19th century, see Figure 7. Cobalt is also a component of the blue smalt pigment [11].

Black pigments are of organic origin and contain predominantly carbon. In two cases, the presence of azurite was detected in the black pigment. Significant peaks of Fe and Zn indicate repaintings made of Mars black (FeO·Fe_2_O_3_), which has been used since the 18th century and has been synthetically produced since 1920, and zinc white (ZnO).

White is also abundant. The presence of Pb peaks in the spectra indicates the use of lead white 2PbCO_3_·Pb(OH)_2_. This is an alkaline calcium carbonate that has been artificially produced since ancient times. Lead white was blended into other colors. From the nineteenth century, it began to be replaced by zinc white ZnO.

**Figure 8:**
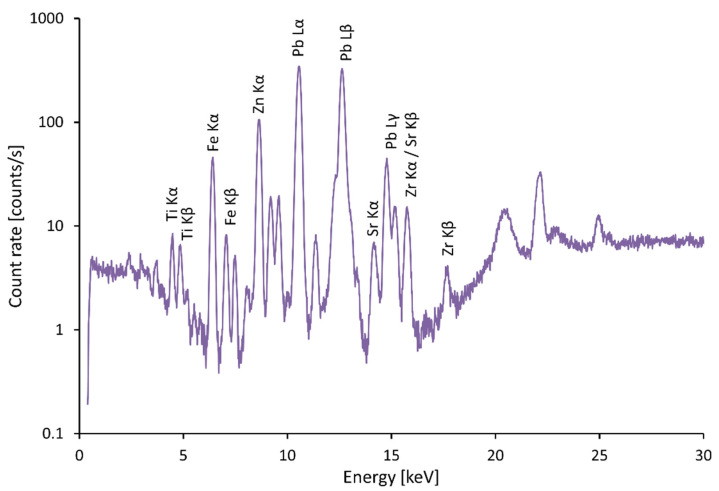
Spectrum of yellow massicot, zinc oxide.

Yellow color replaced gold on coats of arms. The presence of significant lead peaks in the spectra indicates the use of massicot, lead yellow PbO, and the presence of Fe peaks indicates the use of yellow ochers [12]. The presence of Zn peaks in the spectra, e.g., in Figure 8, indicates the use of zinc white and repairs in the 19th and 20th centuries.

**Figure 9:**
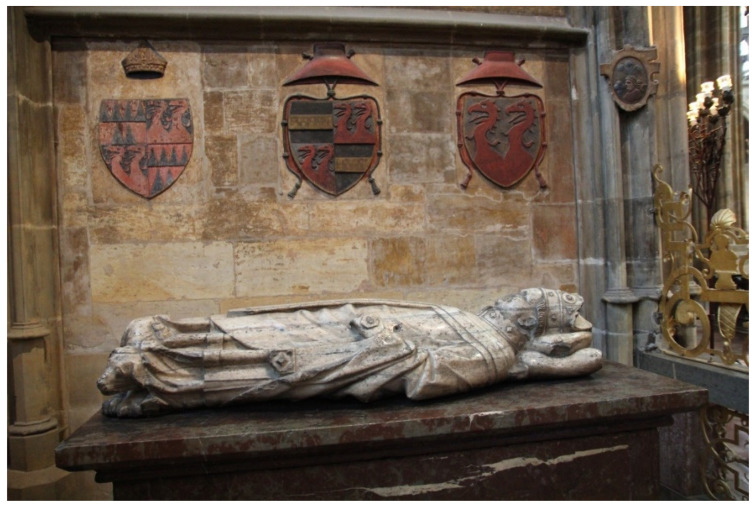
Coats of arms on Vlašim Chapel.

**Figure 10:**
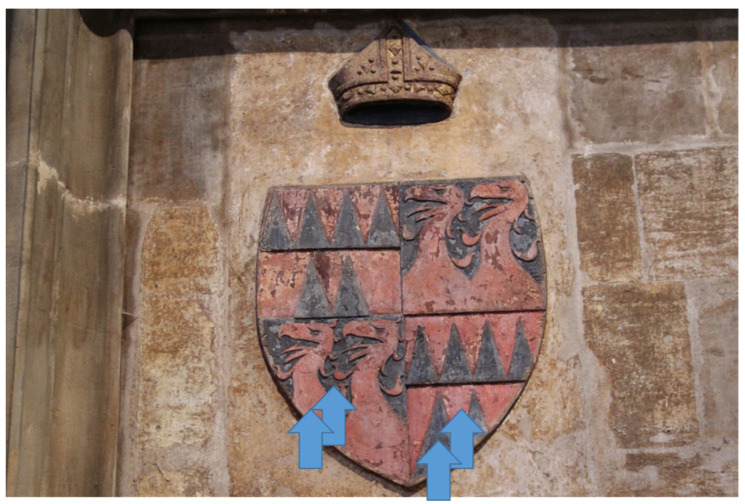
Bishop coat of arms on Vlašim Chapel, arrows indicate measured positions.

The funeral tomb of Archbishop Jan Očko is located in the Vlašim Chapel in St. Vitus Cathedral. Above the tomb, there are three coats of arms that Jan Očko used during his lifetime: the bishop’s coat of arms, the archbishop’s coat of arms, and the family coat of arms with a cardinal’s hat. The compositions of the pigments were measured on 12 locations. Only three colors, red, black, and yellow, were used on the coats of arms in the Vlašim Chapel. See Figure 9 and Figure 10. What has been said about the pigments used on the triforium applies to red and white pigments, but the yellow color is lead-tin yellow.

**Figure 11:**
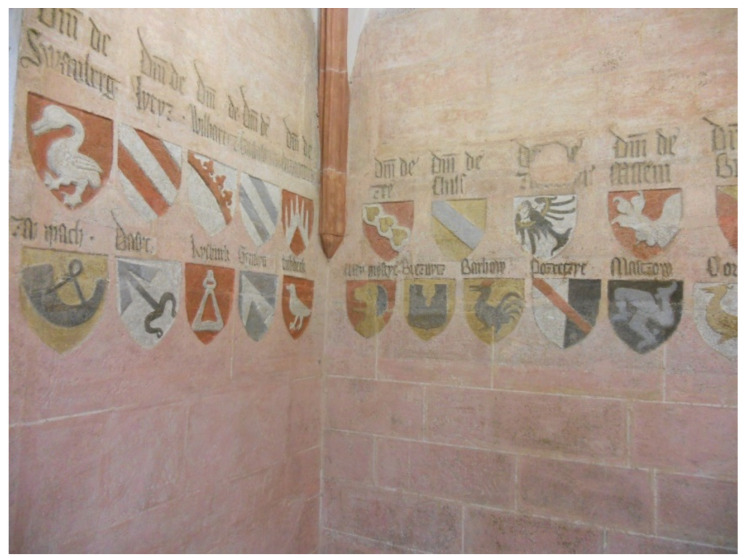
Coats of arms on Lauf Castle.

The same palette of colors as on the signs in St. Vitus Cathedral was used on the signs decorating the Character Hall of Charles IV at Lauf Castle near Nuremberg. We also had the opportunity to measure these coats of arms [13]. Lauf Castle was built by Charles IV in a strategic location east of Nuremberg on territory that then belonged to the Czech Kingdom, in the years 1357–1360. The central area of Lauf Castle is the audience coat of arms hall on the first floor. The emperor had 114 coats of arms carved into the walls of the hall in two rows below him. Above each emblem is a Latin inscription identifying the owner of the coat of arms. This created a unique coat of arms gallery that has no analogs in Central Europe (Figure 11). According to the dating, Charles IV had the decoration created in 1361, after the birth of his son Wenceslas IV. The same pigments were used at Lauf Castle as in St. Vitus Cathedral 20 years later. However, most of the painting on Lauf Castle has been damaged and most of the coat of arms have already been repainted.

## 4. Conclusions

The paper has provided an overview of the results of measurements of pigments used for polychromy of busts and coats of arms in the triforium of St. Vitus Cathedral. The X-ray fluorescence analysis method that was used makes it possible to distinguish medieval pigments from newer repaintings. Medieval master craftsmen used a limited palette of colors, but each color could have different compositions and, thus, different shades. For example, red color could be realized using red lead, cinnabar, or red ocher. Blue color was realized through azurite or smalts. X-ray fluorescence analysis can also determine these differences in the composition of individual colors.

The results of the measurements provide information on which part of the busts and coats of arms have retained their original polychrome and which parts have been restored in the past. The results of the measurements will serve as a source of information for art historians and in possible restorations of triforium decoration.

The results of the measurements on the triforium were compared with the results of measurements in the Vlašim Chapel in St. Vitus Cathedral and the coats of arms in the audience hall at Lauf Castle near Nuremberg.

## Data Availability

Not applicable.
